# A thin layer angiogenesis assay: a modified basement matrix assay for assessment of endothelial cell differentiation

**DOI:** 10.1186/s12860-014-0041-5

**Published:** 2014-12-05

**Authors:** Ashton Faulkner, Robert Purcell, Andrew Hibbert, Sally Latham, Scott Thomson, Wendy L Hall, Caroline Wheeler-Jones, David Bishop-Bailey

**Affiliations:** Comparative Biomedical Sciences, Royal Veterinary College, University of London Royal College Street, London, NW1 0TU UK; Diabetes and Nutritional Sciences Division, School of Medicine, King’s College London, 150 Stamford Street, London, SE1 9NH UK

**Keywords:** Angiogenesis, Endothelial cell, Basement matrix, Mitochondria, PPAR, VEGF

## Abstract

**Background:**

Basement matrices such as Matrigel™ and Geltrex™ are used in a variety of cell culture assays of anchorage-dependent differentiation including endothelial cell tube formation assays. The volumes of matrix recommended for these assays (approximately 150 μl/cm^2^) are costly, limit working distances for microscopy, and require cell detachment for subsequent molecular analysis. Here we describe the development and validation of a thin-layer angiogenesis (TLA) assay for assessing the angiogenic potential of endothelial cells that overcomes these limitations.

**Results:**

Geltrex™ basement matrix at 5 μl/cm^2^ in 24-well (10 μl) or 96-well (2 μl) plates supports endothelial cell differentiation into tube-like structures in a comparable manner to the standard larger volumes of matrix. Since working distances are reduced, high-resolution single cell microscopy, including DIC and confocal imaging, can be used readily. Using MitoTracker dye we now demonstrate, for the first time, live mitochondrial dynamics and visualise the 3-dimensional network of mitochondria present in differentiated endothelial cells. Using a standard commercial total RNA extraction kit (Qiagen) we also show direct RNA extraction and RT-qPCR from differentiated endothelial cells without the need to initially detach cells from their supporting matrix.

**Conclusions:**

We present here a new thin-layer assay (TLA) for measuring the anchorage-dependent differentiation of endothelial cells into tube-like structures which retains all the characteristics of the traditional approach but with the added benefit of a greatly lowered cost and better compatibility with other techniques, including RT-qPCR and high-resolution microscopy.

**Electronic supplementary material:**

The online version of this article (doi:10.1186/s12860-014-0041-5) contains supplementary material, which is available to authorized users.

## Background

Secreted extracellular matrix proteins, purified from the Engelbreth-Holm-Swarm (EHS) tumour, such as Matrigel™ and Geltrex™, are widely used in a variety of cell culture applications [1-4], including the support of primary cell propagation, and anchorage-dependent cell differentiation. These cell differentiation assays include the morphogenesis of endothelial cells (EC) into tube-like structures (tube-formation assay) for the study of *in vitro* angiogenesis [3,5,6], and the differentiation of neural cells in neurite outgrowth assays [1].

The most common assay employed using basement matrix is the EC tube formation assay, thought to represent the differentiation stage of angiogenesis and often being applied as a first-pass screening assay of compounds with potential pro- or anti-angiogenic properties (reviewed in *Staton et al.,* 2009 [7]). To study cell differentiation, a relatively large volume of matrix is usually indicated with recommendations and common usage of matrix in the order of 50-200 μl/cm^2^, depending upon the assay to be employed. As well as increasing the general cost associated with performing these assays this volume of matrix also precludes the use of experimental techniques such as high-resolution/single cell microscopy and requires further cell manipulation (e.g. dispase digestion) [8] in order to extract mRNA for subsequent gene expression analysis. In an attempt to solve these issues we sought to develop a thin layer angiogenesis assay (TLA) that would retain the ability to act as an anchoring and differentiating platform, but could also be used for high definition single cell imaging. Here, we show that spreading low volumes of Geltrex™ basement membrane thinly onto glass or tissue culture plastic enables the use of high-resolution imaging and direct RNA extraction from endothelial cells that are actively engaged in the tube-forming process. This simple assay generates comparable data to those derived from large volume matrix assays, and has a far greater utility in terms of imaging and transcriptional analysis accompanied by a 25–30 fold reduction in relative cost.

## Results

### The human endothelial cell line (EA.hy926) and primary human umbilical vein endothelial cells (HUVEC) undergo differentiation on a thin layer matrix

**Figure 1:**
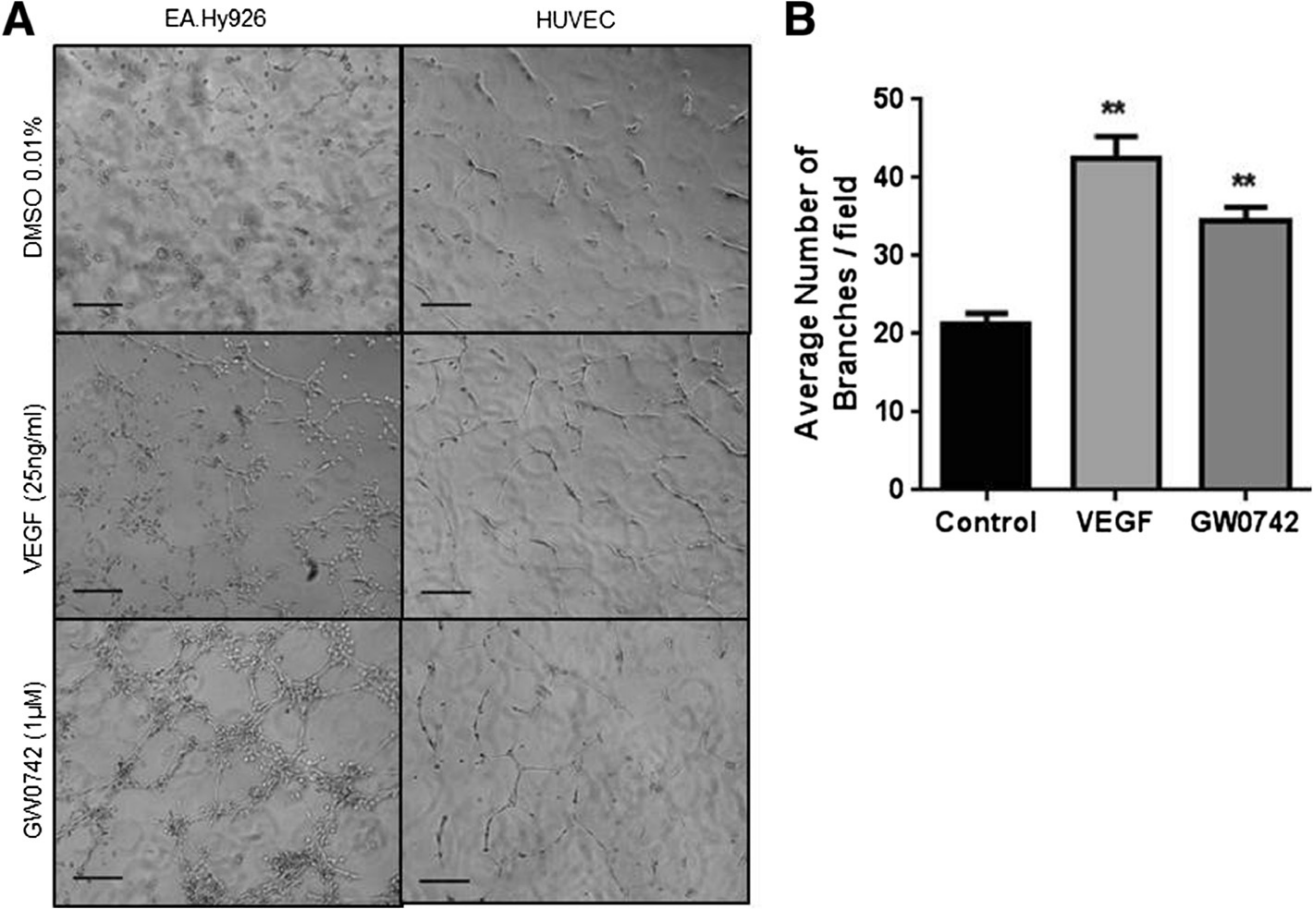
**Human endothelial cells readily differentiate on thin layers of basement matrix spread on glass. (A)** Representative images of EA.hy926 and HUVEC forming tube-like structures after 24 and 16 hours, respectively, when plated onto 10 μl/2 cm^2^ of basement matrix in the presence of VEGF (25 ng/ml) or GW0742 (1 μM). Images were acquired using Leica DMIRB microscope (x10 objective). Scale bar = 200 μm. **(B)** Quantification of tubes formed by HUVEC at 16 hours in the presence of VEGF, GW0742 or DMSO control. Data represent mean (± S.E.M) number of branches/field from n = 5 separate donors. **p < 0.01 vs. DMSO control as determined by repeated measures ANOVA followed by Dunnett’s post analysis.

**Figure 2:**
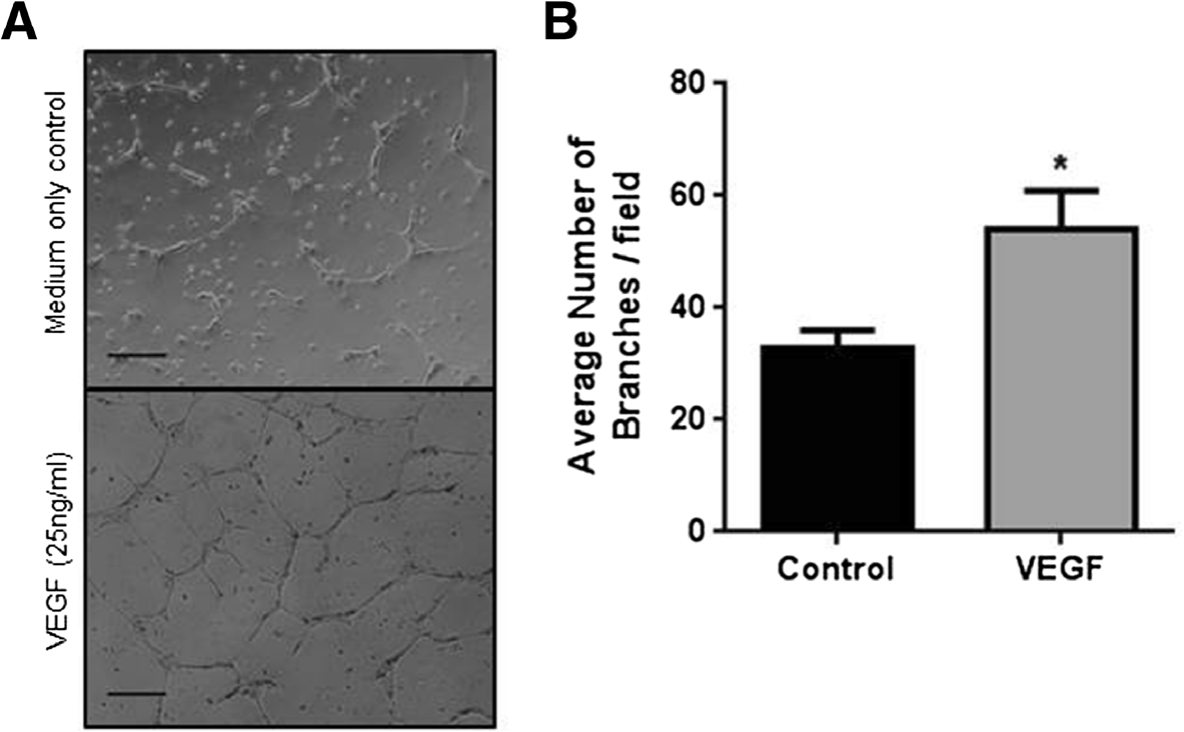
**Human endothelial cells readily differentiate on thin layers of basement matrix spread on tissue culture plastic. (A)** Representative images of HUVEC forming tube-like structures after 16 hours when plated onto 10 μl/2 cm^2^ of basement matrix in the presence of VEGF (25 ng/ml) or medium only control. Images were acquired using Leica DMIRB microscope (x10 objective). Scale bar = 200 μm. **(B)** Quantification of branches formed by HUVEC at 16 hours in the presence of VEGF, or medium control. Data represent mean (± S.E.M) number of branches/field from n = 4 separate donors. *p < 0.05 *vs.* medium control as determined by paired Student’s *t*-test.

10 μl of growth factor-reduced Geltrex™ basement matrix was spread evenly onto glass (13 mm coverslip) using a sterile syringe insert. HUVEC or EA.hy926 formed tube-like structures under non-stimulated conditions (vehicle alone; 0.01% DMSO) and the number of tubes formed was significantly increased in response to the distinct angiogenesis inducers vascular endothelial growth factor (VEGF; 25 ng/ml) and GW0742 (1 μM), a selective PPARβ/δ agonist [3,6] (Figure 1). Similar results were obtained when Geltrex™ matrix was spread directly onto tissue culture plastic with VEGF-stimulated HUVEC showing a clear pro-angiogenic response (Figure 2).

**Figure 3:**
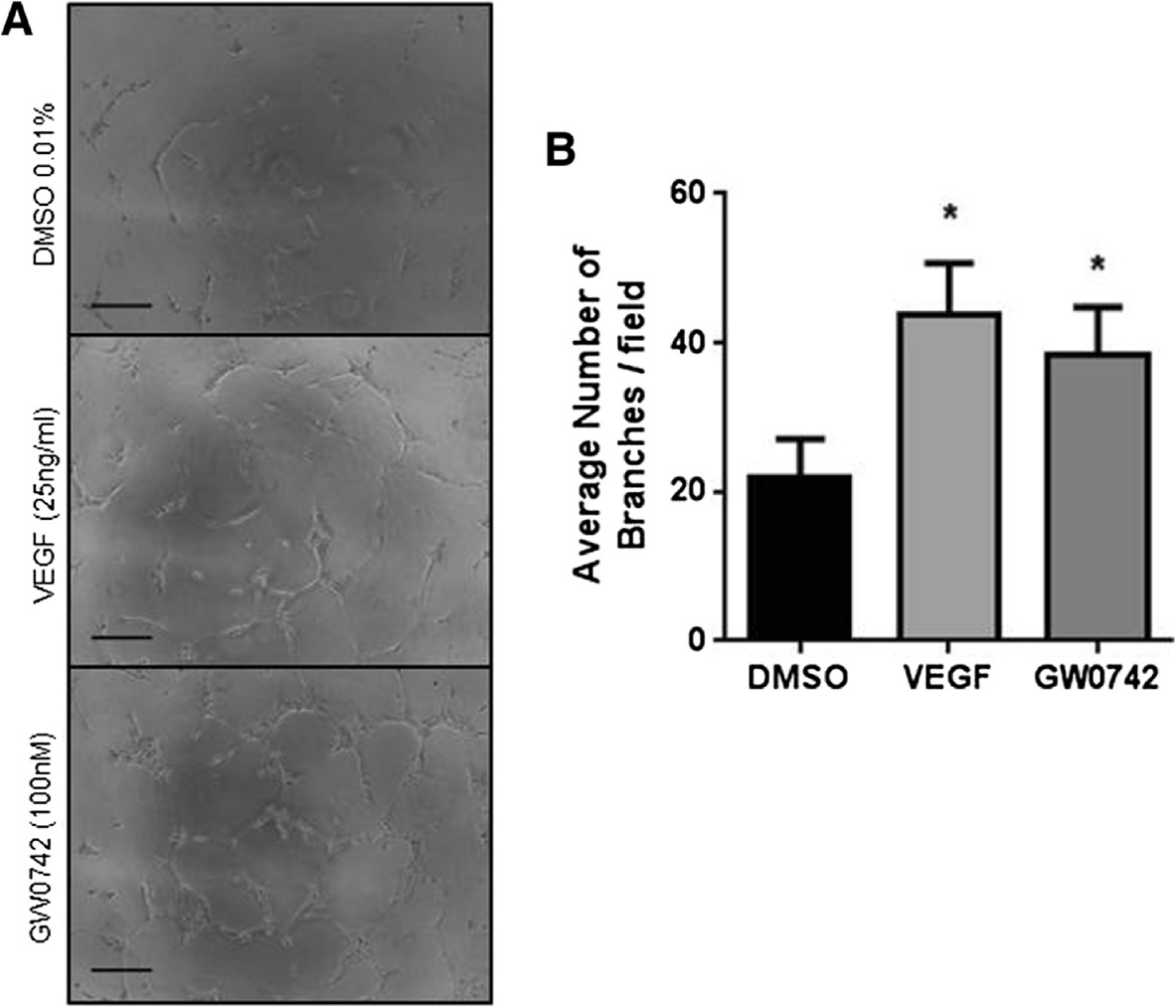
**The thin-layer angiogenesis assay (TLA) can be performed in a 96-well plate format using 2 μl of basement matrix. (A)** Representative images showing HUVEC (25,000 cells/cm^2^) forming tube-like structures after 16 hours when plated onto 2 μl of basement matrix with more tubes forming in the presence of VEGF (25 ng/ml) or GW0742 (100nM). Images were acquired using Leica DMIRB microscope (x10 objective). Scale bar =200 μm. **(B)** Mean number of branches (± S.E.M)/field formed at 16 hours in the presence of VEGF (25 ng/ml), GW0742 (100nM) or DMSO control. *p < 0.05 *vs.* DMSO control as determined by repeated measures ANOVA followed by Dunnett’s post analysis, n = 6 separate donors ran in sextuplet.

An advantage of the traditional angiogenesis assay is scalability, with many research groups performing the assay in a 96-well plate format using 50 μl/well of basement matrix [6,9-11]. To determine if this higher throughput can be achieved with the TLA assay, HUVEC (25,000 cells/cm^2^) were seeded on 2 μl of Geltrex™ basement matrix in the presence of VEGF (25 ng/ml; 16 h) or GW0742 (100 nM; 16 h). These experiments showed that responses to VEGF and GW0742 using the TLA in 96-well plate format (Figure 3) were identical to those measured using the coverslip TLA approach (Figure 1).

**Figure 4:**
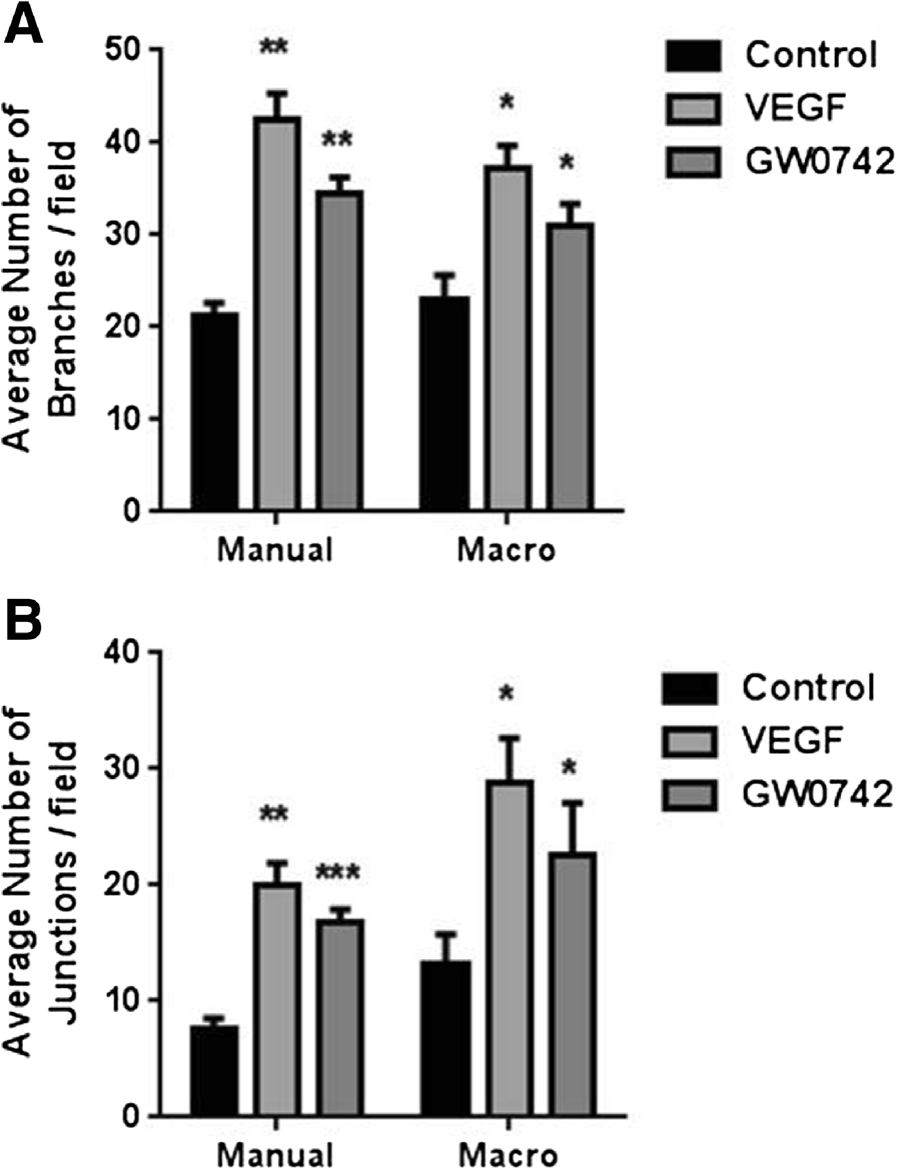
**Comparison of manual versus automated quantitation of angiogenic parameters.** A comparison of **(A)** average number of branches/field and **(B)** average number of junctions/field obtained from the 24-well data acquired from the HUVEC TLA experiments reported in Figure 1 when quantified manually or with the use of the angiogenesis macro for ImageJ. n = 5 independent donors. *p < 0.05 **p < 0.01 ***p < 0.001 vs control as determined by one-way ANOVA followed by Dunnett’s post-analysis test.

A common goal for those routinely using the basement matrix assay for assessing angiogenic potential is the use of automated quantification systems. The use of an automated quantification system (ImageJ Angiogenesis Analyzer) provides qualitatively but not always quantitatively identical data to manual counting (Figure 4; Additional file 1: Table S1).

### The TLA approach is qualitatively similar to the traditional thick layer approach

**Figure 5:**
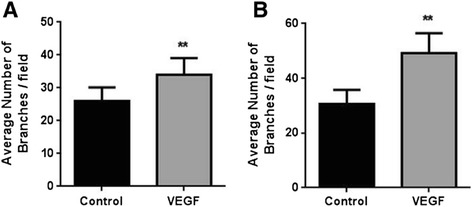
**Direct comparison of the thin layer angiogenesis (TLA) assay with standard thick layer assay.** Comparison of the average number of branches/field (manual count) formed by HUVEC (25,000 cells/cm^2^) in the presence or absence of VEGF (25 ng/ml) in the standard thick layer assay **(A)** and the TLA assay **(B)**. **p < 0.01 vs. control as determined by student’s paired *t*-test.

To make a direct comparison of the TLA approach with the traditional thick layer, HUVEC were stimulated to undergo tubulogenesis in the presence or absence of VEGF (25 ng/ml) on either 2 μl/well or 50 μl/well of Geltrex™ in a 96-well format. VEGF induced a similar pro-angiogenic effect on HUVEC whether on a standard thick or TLA basement matrix (Figure 5; Additional files 2: Movie S1A and S1B).

### Differential interference contrast (DIC) and confocal microscopy of differentiated endothelial cells

**Figure 6:**
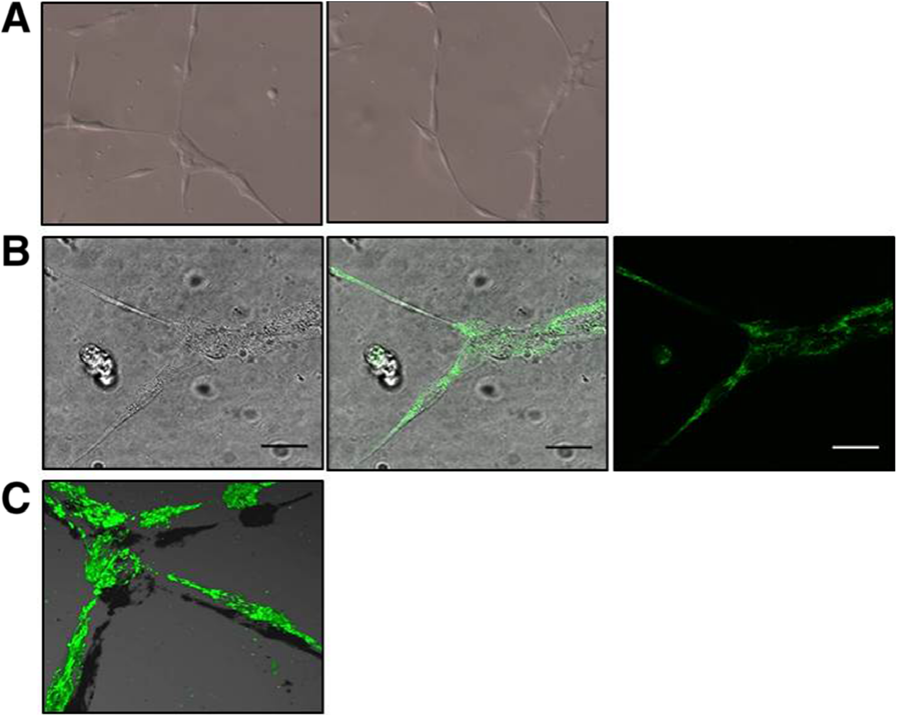
**High contrast images of differentiated HUVEC in the TLA assay by Differential Interference Contrast (DIC) and confocal fluorescence microscopy.** HUVEC stimulated to form tube-like structures with 25 ng/ml VEGF on a 35 mm glass-bottom dish covered with 10 μl matrix is compatible with DIC (x20 objective) **(A)** and confocal microscopy (x63 objective); Scale bar = 25 μm **(B)**. Mitochondria (green) were stained using MitoTraker green (200nM). For 3D imaging **(C)** of the mitochondrial network, z-stacks were obtained at x40 magnification and re-constructed using Volocity software.

Unlike cell monolayers, the traditional tube formation assay with its large matrix volume has greatly reduced microscopic working distances, making single cell analysis highly challenging. Using HUVEC stimulated with VEGF (25 ng/ml; 16 h) in 35 mm glass-bottom plates coated with 10 μl of Geltrex™, differential interference contrast (DIC) images were acquired using a Zeiss Axiovert 135 microscope (×20 objective). As shown in Figure 6A the TLA is clearly compatible with use of this high contrast technique.

As proof-of-principle for live single cell and confocal imaging we chose the well-established mitochondrial-specific dye MitoTracker [12]. VEGF-stimulated HUVEC (25 ng/ml; 16 h) were incubated for 40 min with MitoTracker green (200nM). Live mitochondrial dynamics (over 10 min; ×63 objective) were recorded within the tube-like structures (Figure 6B and Additional file 3: Movie S2). In addition to single cell-live imaging, the 3D mitochondrial network in differentiated endothelial cells was reconstructed following z-stack deconvolution (×40 objective) using a Leica SP5 confocal microscope (Figure 6C and Additional file 4: Movie S3).

**Figure 7:**
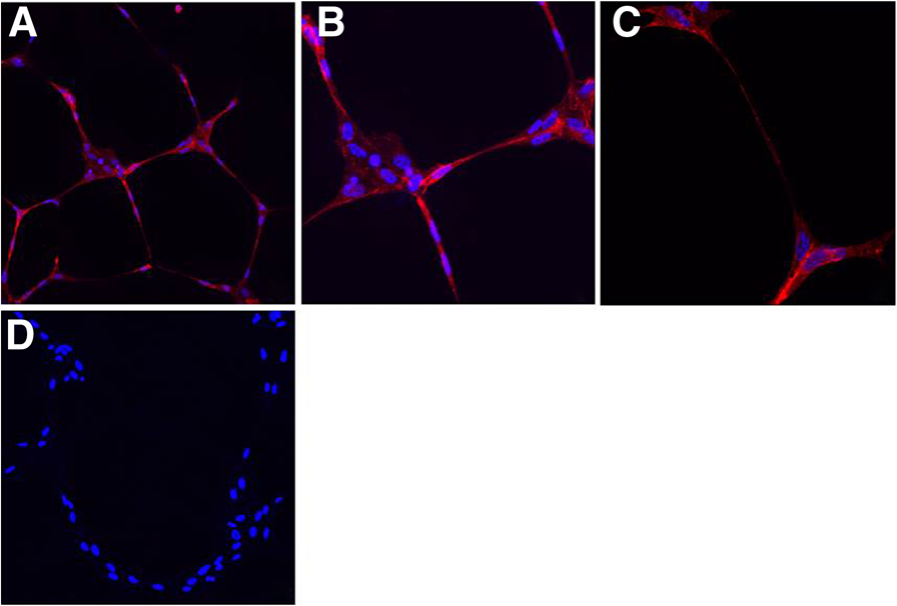
**Confocal immunofluorescence of CD31 in HUVEC differentiated in the TLA assay.** Tube-like structures formed by HUVEC in the presence of 25 ng/ml VEGF for 16 h at **(A)** x20, **(B)** x40 and **(C)** x63 magnification with CD31 labelled in red and nuclei in blue. **(D)** represents secondary antibody control.

To demonstrate confocal imaging of fixed cells, HUVEC differentiated using the TLA approach in chamber slides (VEGF 25 ng/ml; 16 h) were fixed with 4% PFA and stained for the common endothelial cell marker CD31 (PECAM) (Figure 7 and Additional file 5: Movie S4).

### Direct RNA extraction from HUVEC in the TLA assay for use in RT-qPCR

**Figure 8:**
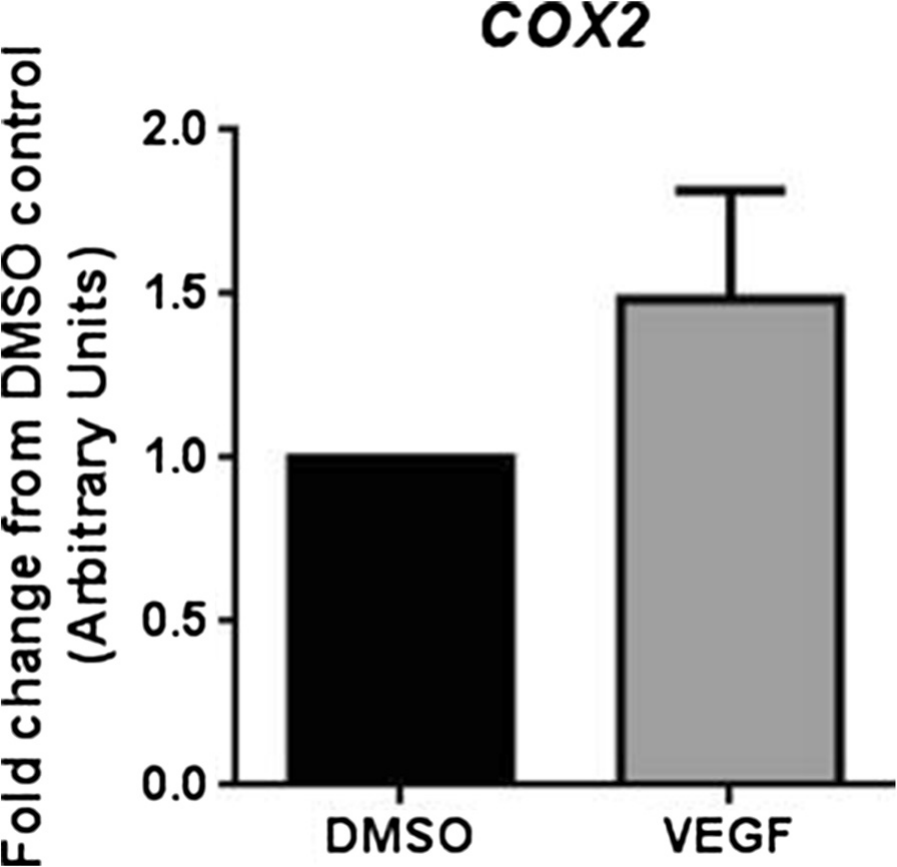
**Direct RNA extraction and RT-qPCR analysis of HUVEC immediately after the TLA assay.** Fold relative COX-2 mRNA to GAPDH expression in VEGF-treated HUVEC undergoing differentiation in 24-well plates in the TLA assay. RNA was directly extracted from the cells and targets measured by Taqman RT-qPCR; n = 4 separate experiments.

Total RNA was extracted from HUVEC differentiated into tubes using the TLA assay following exposure to VEGF (25 ng/ml) without prior cell isolation according to the manufacturer’s recommended protocol (Qiagen Total RNA Extraction kit). Subsequent standard RT-qPCR analysis readily detected GAPDH and COX-2 expression (previously shown to increase in HUVEC in response to VEGF treatment [6]). COX-2 expression in the TLA was not significantly induced relative to GAPDH at 16 h in VEGF stimulated tube-forming HUVEC (Figure 8 and Additional file 6: Figure S2).

## Discussion

The use of a basement matrix in the study of anchorage-dependent differentiation is common place in many research laboratories, with the endothelial cell tube formation assay being the most widely used *in vitro* assay for the study of angiogenesis. The smallest volume of matrix used in this assay to date is 15 μl/cm^2^ (30 μl per well of a 24-well plate) [13] which is more cost effective than the recommended volume of 50 μl/cm^2^ (100 μl per well of a 24-well plate) but created a meniscus that promoted cell pooling [14]. In theory, if the matrix layer becomes thin enough, significant meniscus formation and subsequent cell pooling would be overcome. Here, we demonstrate that the use of as little as 5 μl/cm^2^ (10 μl per well of a 24-well plate) is sufficient to support the anchorage-dependent differentiation of endothelial cells into tube-like structures without meniscus formation and cell pooling. Using this thin layer approach we demonstrate that the assay can be reproducibly performed on glass or on plastic and that quantification of tube-forming capacity shows the expected pro-angiogenic responses to VEGF and the PPARβ/δ agonist GW0742 (Figures 1, 2, 3 and 5), consistent with published data from our and other laboratories using a traditional matrix volume [3,6,15,16] and re-confirmed in side-by-side comparisons in this paper (Figure 5). Moreover, the TLA method may provide a platform for more stable tube formation with less network breakdown (Additional file 2: Movie S1). The reason for this is not clear but may represent a further potential advantage over the traditional thick layer approach.

A common problem with the standard assay is its compatibility with automated analysis systems due to loss of focus, predominantly as a consequence of meniscus formation. With the TLA method reducing meniscus formation we sought to identify if automated analysis using the ImageJ Angiogenesis Analyzer freeware was compatible with this thin layer approach. Automated analysis was qualitatively (if not always quantifiably) similar to manual counting (Figure 4), and can therefore (with this caveat) be readily applied to the TLA method.

An additional benefit of using the TLA assay in a 24-well format is the ability to directly extract mRNA from the tube-forming cells for gene expression analysis. Traditionally, due to the large volume of gel present this has been achieved by isolating the cells from the matrix (e.g. by dispase digestion) which requires subjecting the cells to additional chemical treatment and centrifugation [8,17]. Accordingly, most studies investigate gene expression in endothelial cell monolayers and correlate the results with outcomes from the functional tube formation assay [3,18,19]. With the highly reduced gel volume, the TLA approach presented here negates the need for cell detachment from the matrix, thus allowing direct extraction of RNA from cells which are undergoing or have completed tubulogenesis. This modification therefore makes the molecular analysis less time-consuming as well as circumventing the difficulties associated with extrapolating between data derived from cell monolayers *versus* differentiating endothelial cells. The ability to increase throughput is an advantage for any assay, particularly when it may be desirable to screen the effects of a number of different compounds. In this respect we show that the TLA methodology can be used in a higher throughput format in 96-well plates using 2 μl of matrix/well (Figure 3).

The ability to obtain high-resolution images from a convenient low-cost *in vitro* assay is highly desirable. We show here that one of the advantages of the TLA assay with its greatly reduced working distances is the use of high-resolution microscopy, including DIC and confocal, that allows a more accessible means of studying cellular and intracellular processes, demonstrated here by the immunofluorescent staining of CD31. Moreover, with the growing interest in the role played by cell organelles such as the mitochondria in the angiogenic process [20,21], we have successfully monitored the mitochondrial network and dynamics during endothelial cell tubulogenesis. To our knowledge we are the first to show such high-resolution images of live mitochondria within developing tubules.

## Conclusions

The use of a basement matrix in the study of anchorage-dependent differentiation is common place in many research laboratories, with the endothelial cell tube formation assay being the most widely used *in vitro* assay for investigating the angiogenic functions of endothelial cells. Current recommended matrix volumes make this an expensive assay, preclude the use of more advanced imaging techniques, and limit the ease with which molecular data can be acquired. We have described a modified method for this basement matrix assay which provides all the necessary support required for differentiation, generating results which are similar to those obtained using the traditional approach. In conclusion, our thin layer angiogenesis assay makes for the ideal first-pass angiogenesis screening assay with a substantially lowered cost (25–30 fold) compared to commonly used similar *in vitro* angiogenesis assays, whilst at the same time maximising high through put testing, and gives the user greatly reduced working distances that allows, for the first time with this assay, single cell, high resolution imaging of actively differentiating cells.

## Methods

### Cell culture

EA.Hy926 cells were cultured in Dulbecco’s Modified Eagles Medium (DMEM) (Sigma Aldrich, Gillingham, UK) supplemented with 10% foetal bovine serum (FBS) and 1% penicillin/streptomycin (Pen/Strep) at 37°C, 5% CO_2_. Unless stated, experiments were carried out in serum-free DMEM (1% pen/strep). Human Umbilical Vein Endothelial Cells (HUVEC) were isolated from donated cords as previously described [6] and cultured under standard conditions (37°C, 5% CO_2_) in M199 supplemented with 20 μg/ml endothelial cell growth factor (ECGF) and 20% FBS. Cells were used at passage 2. Unless stated, HUVEC experiments were carried out in M199 (1% FBS; 1% pen/strep).

### Tube formation assay

To induce thin-layer angiogenesis (TLA) two approaches were taken: *1) Coverslip approach:* 10 μl of basement matrix (Geltrex™, Life Science Technologies) (kept continuously on ice) was placed on the centre of a 13 mm glass coverslip and spread evenly with the use of a cell scraper, providing a thin layer covering the surface of the coverslip. Each coverslip was subsequently transferred into the well of a 24-well plate (pre-cooled on ice) with the use of forceps. This process was repeated for each well before the gel was allowed to set at 37°C for 30 minutes. *2) Direct approach:* 10 μl of basement matrix was placed directly into the centre of each well of a 24-well plate and spread evenly with the rubber end of a sterile 1 ml syringe insert. The plate was placed at 37°C for 30 minutes to allow the gel to set. After a 1 h serum-starvation in experimental media, cells were plated at a density of either 25,000 cells/cm^2^ (HUVEC) or 100,000 cells/cm^2^ (EA.Hy926) [3] and incubated for either 16 hours (HUVEC) [3] or 24 hours (EA.Hy926) in the appropriate experimental medium supplemented with either 25 ng/ml VEGF, 1 μM GW0742 or 0.01% DMSO vehicle control (in duplicate). Phase-contrast images (4 images/well) were acquired using a Leica DMIRB inverted microscope (×10 magnification) and the mean number of branches/high powered field counted manually using imageJ software to form each n-number from each donor. For clarity, the structures routinely considered as branches for quantification purposes are highlighted in the accompanying Additional file 7: Figure S1. For automated quantification, images were analysed using the angiogenesis macro for ImageJ.

To test scalability, the TLA assay was also performed in 96-well plates, using wells coated with 2 μl/well of basement matrix, spread evenly with the use of an insert of a sterile Eppendorf 0.5 ml combitip (cat #: 0030089421). HUVEC were seeded at a density of 25,000 cells/cm^2^ in experimental medium supplemented with 25 ng/ml VEGF, 100nM GW0742 or DMSO (vehicle) control (in sextuplet), and incubated at 37°C/5% CO_2_ for 16 hours. Phase-contrast images were acquired using a Leica DMIRB microscope (×10 magnification) at the centre of each well and the number of branches counted manually following identification with ImageJ software.

For the comparison of the TLA assay with the traditional assay approach, HUVEC (25,000 cells/cm^2^) were seeded onto either 2 μl/well (TLA) or 50 μl/well (traditional) of matrix in 96-well plates in the presence or absence of VEGF (25 ng/ml) and left to form tubes for 16 hours. Images were acquired using the Leica DMIRB inverted microscope as described above and average number of branches/high power field quantified manually using ImageJ software. For live imaging of cell differentiation, images were acquired every 30 minutes using Leica SP5 confocal microscope (×10 objective).

### Microscopy

HUVEC (25,000 cells/cm^2^) were seeded into a 35 mm glass-bottom dish in which the glass area had been pre-coated with 10 μl of Geltrex™ basement membrane and cells induced to undergo tube formation with VEGF (25 ng/ml; 16 h). Medium was then replaced with fresh M199 containing 200nM MitoTraker green and the cells incubated for a further 40 min at 37°C. Dye-containing medium was then replaced with fresh M199 prior to imaging.

Differential interference contrast (DIC) images were obtained using a Zeiss Axiovert 135 microscope fitted with a ×20 objective. Confocal fluorescence images of mitochondria were obtained using a Leica SP5 confocal microscope fitted with a water emersion (×63) objective. For z-stack series, images were obtained using a ×40 objective and 3D images constructed using Volocity software (PerkinElmer; version 6.3.1).

### Immunocytochemistry

The TLA assay was performed in 8-well chamber slides (LabTek) with HUVEC (25,000 cells/cm^2^) seeded onto 3 μl of Geltrex basement matrix in the presence or absence of 25 ng/ml VEGF. Tube-like structures were allowed to form for 16 h. After the careful removal of media, cells were washed with PBS and then fixed with 4% PFA for 10 min. Cells were permeabilised with 0.1% Triton-x 100, blocked in 3% BSA 1% goat serum for 30 min, and CD31 detected using anti-CD31 rabbit polyclonal primary antibody (1:250; 1 h) (Santa Cruz) and Alexa fluor 568 goat anti-rabbit secondary antibody (30 min; Life Technologies). Cells were then mounted with DAPI-containing mounting media (Sigma, UK). Images were acquired with a Leica SP5 confocal fluorescence microscope. 3D images were constructed using Volocity software (PerkinElmer; version 6.3.1).

### RNA extraction and cDNA synthesis

Total RNA was extracted using the on-column Qiagen (Manchester, UK) Total RNA Extraction kit as per the manufacturer’s instructions. Briefly, media was removed from the wells by careful pipetting and any dead cells washed away with PBS. The plate was observed under a bright-field microscope to check for any disruption of cells. If no disruption was evident, 175 μl of lysis buffer was added to each well and the resulting lysate was pooled for each treatment to achieve a final volume of 350 μl of lysate per treatment. Lysates were put through a genomic spin column to remove as much genomic DNA contamination as possible. All remaining steps for RNA purification was performed by strict adherence to the manufacturer’s instructions. RNA was eluted into 40 μl of nuclease-free H_2_O and concentrations were determined using a NanoDrop-1000. cDNA was synthesised, as per manufacturer’s guidelines using SuperScript™ II Reverse Transcriptase obtained from Invitrogen. Briefly, isolated RNA (equal amount per treatment) was combined with 1 μl of Oligo(dT) (500 μg/ml) and 1 μl dNTP mix (10 mM each). The mixture was heated to 65°C for 5 minutes. Subsequently, a master mix consisting of 5X first-strand buffer, 0.1 M DTT and SuperScript™ II Reverse Transcriptase (100U/reaction) was added to the mixture to give a final reaction volume of 20 μl. The mixture was then heated to 42°C for 50 minutes before the reaction was inactivated by heating to 72°C for 15 minutes. cDNA was stored at −20°C until further analysis.

### Quantitative RT-PCR

Quantitative RT-PCR was performed using Taqman probe-based technology (Applied Biosystems) for cyclo-oxygenase (COX)-2 (Cat # Hs01573477_g1) and the internal control gene GAPDH (Cat # Hs02758991_g1). For each gene, a primer-probe master-mix and nuclease-free H_2_O was prepared and pipetted into each well of a 96-well PCR plate (18 μl/well). 2 μl of cDNA sample was then added to the appropriate wells. A minus cDNA template (H_2_O) control was run for each gene. All reactions were performed in duplicate. The PCR reaction was run on a Chromo-4 machine using Opticon software under the following conditions: 50°C for 2 min, 95°C for 10 min followed by 40 cycles of 95°C for 15 seconds and 60°C for 30 seconds with a plate read at the end of each cycle. C(t) values were obtained at a cycle threshold value of 0.1. Results were normalised to GAPDH and analysed using the ΔΔCT method.

### Statistical analysis

All data are expressed as mean ± SEM. Statistical differences were assessed by paired Student’s *t*-test or repeated measures one-way ANOVA with Dunnett’s post-analysis where appropriate. *p* < 0.05 was considered statistically significant.

## Additional files

Additional file 1: Table S1. Common parameters measured in the tube-formation assay as assessed by automated analysis software. Results of the automated analysis macro for ImageJ of tube-like structures formed by HUVEC seeded on to Geltrex-covered (10 μl) coverslips in 24-well plates in the presence of VEGF (25 ng/ml), GW0742 (1 μM) or DMSO (0.01%) for 16 h. **p* < 0.05 vs. control as determined by paired Student’s *t*-test. Additional file 2: Movie S1. Live monitoring of HUVEC dynamics when plated on Geltrex basement matrix – TLA versus standard assay. HUVEC dynamics over 16 h when plated on Geltrex basement matrix in either the TLA assay (2 μl/well; A) or standard tube-formation assay (50 μl/well; B) in 96-well plates stimulated to undergo tubulogenesis with 25 ng/ml VEGF. Images were acquired using the Leica SP5 confocal microscope (x10 objective). Additional file 3: Movie S2. Live confocal imaging of mitochondria in HUVEC tube-like structures from the TLA assay. Mitochondrial dynamics over 10 min (green) in tubes formed by HUVEC induced to undergo tubulogenesis by VEGF (25 ng/ml; 16 h). Images were obtained using Leica SP5 confocal microscope (x40 objective). Additional file 4: Movie S3. 3D imaging movie of mitochondria in HUVEC tube-like structures from the TLA assay. 3D reconstruction of tubes with visible mitochondria (green) formed by HUVEC induced to undergo tubulogenesis by VEGF (25 ng/ml; 16 h). Additional file 5: Movie S4. 3D imaging movie of CD31 (PECAM) in HUVEC tube-like structures from the TLA assay. 3D reconstruction of HUVEC stimulated to undergo tubulogenesis by VEGF (25 ng/ml) showing positive expression of CD31 (red) at the cell membrane. Images were acquired using Leica SP5 confocal microscope (x40 objective). Additional file 6: Figure S2. RT-qPCR can be successfully performed on RNA extracted directly from HUVEC undergoing tubulogenesis in the TLA assay. Example of amplification plots demonstrating the successful detection and amplification of glyceraldehyde phosphate dehydrogenase (GAPDH) **(A)** and cyclooxygenase- 2 (COX-2) **(B)** by Taqman RT-qPCR and associated C(t) values, n = 4 separate experiments. Additional file 7: Figure S1. Manual quantification of tubes using ImageJ software. Example of an original image of tube-like structures formed by HUVEC in the presence of VEGF (25 ng/ml; 16 h) **(A)** and after manual quantification using ImageJ **(B)** with Red markers highlighting individual branches.
