# Supplementary material for: A thin layer angiogenesis assay: a modified basement matrix assay for assessment of endothelial cell differentiation
Source: BMC Cell Biol. 2014 Dec 5;15:41. doi: 10.1186/s12860-014-0041-5 (PMC4263020; doi:10.1186/s12860-014-0041-5)
Supplement: Additional file 1: Table S1. — Common parameters measured in the tube-formation assay as assessed by automated analysis software. Results of the automated analysis macro for ImageJ of tube-like structures formed by HUVEC seeded on to Geltrex-covered (10 μl) coverslips in 24-well plates in the presence of VEGF (25 ng/ml), GW0742 (1 μM) or DMSO (0.01%) for 16 h. *p < 0.05 vs. control as determined by paired Student’s t-test. [file 12860_2014_41_MOESM1_ESM.docx]

|  | Control | VEGF | GW0742 |
| --- | --- | --- | --- |
| Branches | 23 | 37* | 31* |
| Branch Length (Pixels) | 90.99 | 90.88 | 99.71 |
| Nodes | 45 | 99* | 77* |
| Junctions | 13 | 29* | 23* |
