# Supplementary figures and images for: A thin layer angiogenesis assay: a modified basement matrix assay for assessment of endothelial cell differentiation
Source: BMC Cell Biol. 2014 Dec 5;15:41. doi: 10.1186/s12860-014-0041-5 (PMC4263020; doi:10.1186/s12860-014-0041-5)

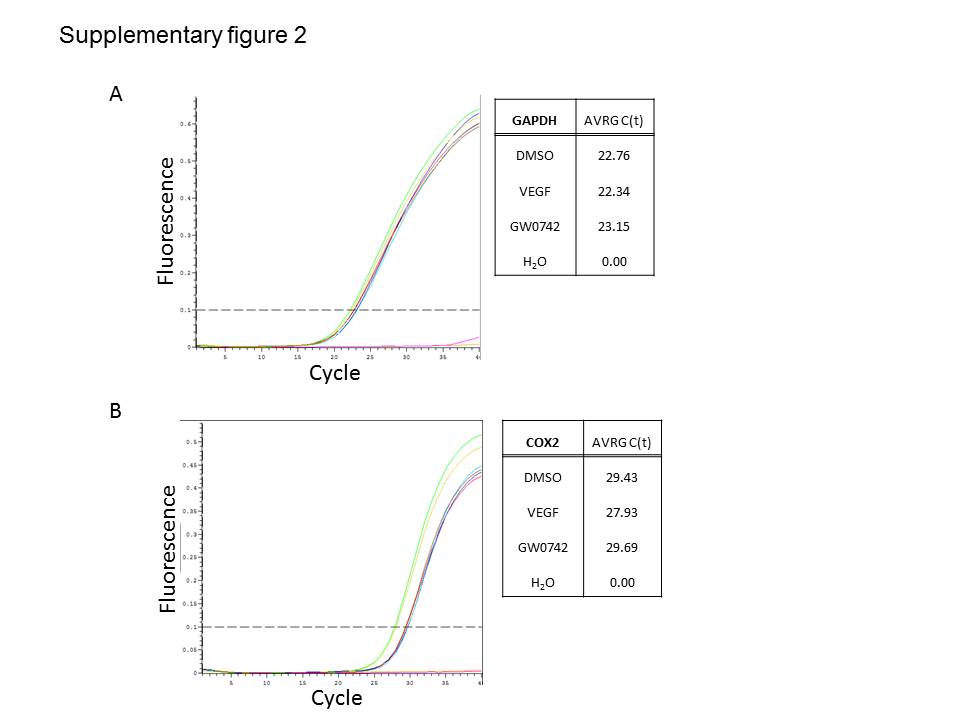

Supplement: Additional file 6: Figure S2. — RT-qPCR can be successfully performed on RNA extracted directly from HUVEC undergoing tubulogenesis in the TLA assay. Example of amplification plots demonstrating the successful detection and amplification of glyceraldehyde phosphate dehydrogenase (GAPDH) (A) and cyclooxygenase- 2 (COX-2) (B) by Taqman RT-qPCR and associated C(t) values, n = 4 separate experiments. [file 12860_2014_41_MOESM6_ESM.jpeg]

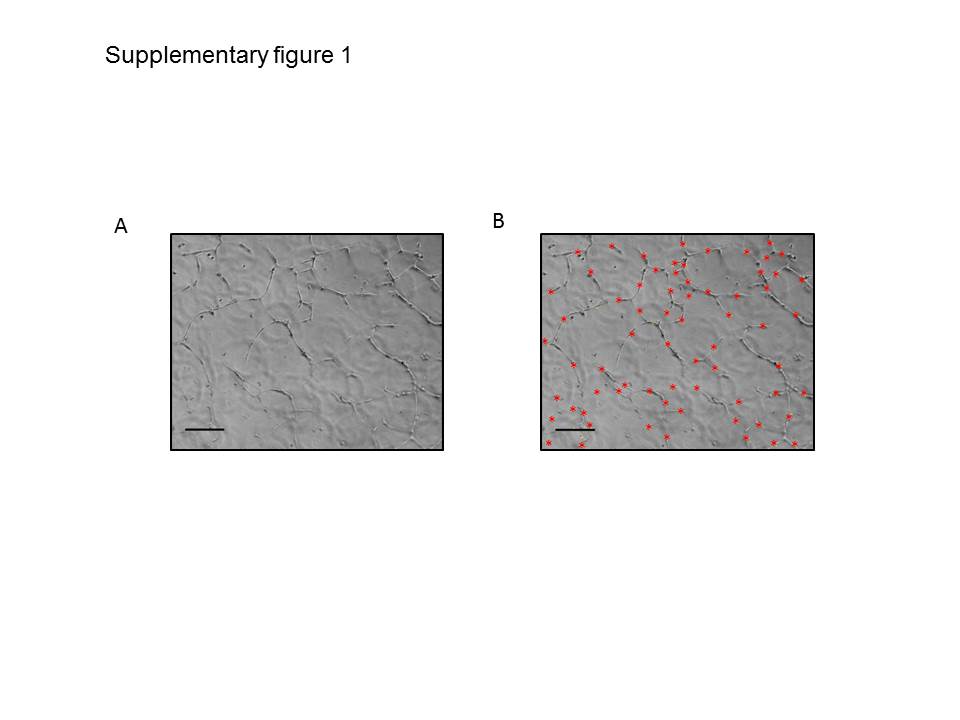

Supplement: Additional file 7: Figure S1. — Manual quantification of tubes using ImageJ software. Example of an original image of tube-like structures formed by HUVEC in the presence of VEGF (25 ng/ml; 16 h) (A) and after manual quantification using ImageJ (B) with Red markers highlighting individual branches. [file 12860_2014_41_MOESM7_ESM.jpeg]
